# Cone-beam computed tomographic analysis of root canal morphology of permanent mandibular incisors - Prevalence and related factors

**DOI:** 10.12669/pjms.38.6.5426

**Published:** 2022

**Authors:** Reham Ahmad Alaboodi, Swati Srivastava, Muhammad Qasim Javed

**Affiliations:** 1 Reham Ahmad Alaboodi, BDS, Intern, Department of Conservative Dental Sciences, College of Dentistry, Qassim University, Buraydah, Qassim, Saudi Arabia; 2 Swati Srivastava, MDS, Associate Professor, Department of Conservative Dental Sciences, College of Dentistry, Qassim University, Buraydah, Qassim, Saudi Arabia; 3 Muhammad Qasim Javed, Assistant Professor, Department of Conservative Dental Sciences, College of Dentistry, Qassim University, Buraydah, Qassim, Saudi Arabia

**Keywords:** Apical third, Central mandibular incisor, Cone beam computed tomography, Lateral mandibular incisor, Oval canals, Root canal morphology

## Abstract

**Objectives:**

To investigate the prevalence of additional canals and the occurrence of oval canals in apical third area of mandibular permanent incisors of Saudi sub-population.

**Methods:**

This study was conducted from November 2020 to May 2021 at College of Dentistry, Qassim University. For the investigation purpose of this study, 314 scans were analyzed within the age limits of 13 to 70 years. The root canal morphology, presence of oval canals, number of roots, and prevalence of various canal configurations based on age, gender and bilateral symmetry were recorded. The obtained data was statistically analyzed using SPSS software.

**Results:**

The mandibular central incisors (CI) exhibited, significant difference between Type-I, II, III and IV canal configurations and Type-I, II, III and V canal configurations (p < 0.05). For the mandibular lateral incisor (LI), significant difference was found between Type-I, II, III, IV and VII canal configurations (p < 0.05). The cumulative prevalence of oval canals in mandibular incisors was found to be 46.6%. For both mandibular CI and LI, the prevalence of Type-I canals was significantly higher in males as compared to females (p < 0.05). Conversely, significantly higher prevalence of Type-III canals was noted for females as compared to males (p < 0.05). No significant difference was found in the prevalence of different canal configurations on the left and the right side of the mouth.

**Conclusion:**

In this study, multiple canals were prominently recognized with Type-III mandibular incisors dominating this feature. Oval canals were predominantly found in single canal especially Type-III. This research suggests variability in canal morphology among different populations. Knowledge of these aberrant canal anatomies is useful for the clinician to achieve a favorable endodontic outcome.

## INTRODUCTION

Profound knowledge of root canal anatomy is a basic prerequisite for successful root canal treatment.^1^-^4^ This understanding of both preoperative and intraoperative evaluation of atypical anatomy, aids the clinician in locating and cleaning canals efficiently.^5^

It was thought that single rooted teeth had single canals like mandibular incisors. However, the discovery of multiple canals in mandibular central and lateral incisors by Rankine and Henry in 1965 sparked further research and confirmed great variations in different human populations.^6^ A dentinal bridge is often present in the pulp chamber of mandibular incisors. This leads to formation of two canals which ultimately end up in one apical foramen (Vertucci Type-III). Although, a single canal is present in the apical third area, it is not uncommon to find that they are usually oval shaped.^7^ Two canals in mandibular incisors has been noted to be in the range of 0.3% to 45.3%.^8^,^9^ Moreover, mandibular incisors have various canal configurations which may alter significantly with different race, ethnicity and gender.^10^

To address the need of identifying canal anatomy, the use of well-established CBCT imaging has become prominent in recent years. It can be used for both quantitative and qualitative analysis in three-dimensions irrespective of high bone density structures in the surrounding area.^10^

A literature search showed a lack of data on the anatomy of mandibular incisors in Saudi Arabian population. Currently, there is restricted data with respect to the true prevalence of different canal configurations of mandibular incisors in Saudi sub-population using CBCT. Our primary outcome was to assess the prevalence of more than one canal in mandibular incisors using CBCT in Al-Qassim sub-population. The secondary outcome was to examine the oval canals prevalence in apical third area of mandibular incisors. The tertiary outcome was to correlate it with other criteria like gender, age and bilateral prevalence.

## METHODS

The institutional review board (Ref.: EA/F-2019-3007 dated Oct. 27, 2019) at College of Dentistry, Qassim University granted approval to the study. The study was conducted from November 2020 to May 2021. The calculation of sample size was done by utilizing 95% confidence interval with a precision of 5%. GALILEOS Comfort CBCT machine (Dentsply- Sirona Dental Systems, Montagematerial, Galileos, SK, Bensheim, Germany) that utilises 85 kVp voltage; 5-7 mA current; field of view 15X15X15 cm^3^; isotropic voxel size 0.3-0.15 mm, was used. Overall, CBCT scans of 314 patients in the age group of 13 to 70 years which were taken from February 2017 to January 2020, were analyzed in the study.

###  The inclusion criteria

Good quality CBCT images with the scans showing the complete unobstructed clear view of the fully erupted and bilaterally present permanent mandibular incisors.

###  The exclusion criteria

Previously treated teeth, open apex, orthodontic treatment history and presence of pathologies.

The CBCT images were assessed by utilizing the scanner’s proprietary software (Sidexis XG 3D Viewer; Germany). The images were adjusted by using software’s image processing tool to ensure optimal visualization. The assessment of mandibular incisors was done in coronal, sagittal, and axial planes. Brightness and contrast of the images were adjusted to enhance measuring procedure. Based on gender, the scans were divided into three age group. i.e., 13-30 years, 31-50 years, and 51-70 years.

The following factors were noted: **a)** The roots number. **b)** The root canal classification according to Vertucci.^11^ **c)** The prevalence of oval canals in Vertucci Type-I and Type-III canal configurations in apical 3rd. **d)** The prevalence of various canal configurations based on age, gender and bilateral symmetry.

The analysis of CBCT scans were done by two experienced observers: An Endodontist with nine years of experience and an Oral Radiologist. The observers were calibrated with the use of viewing software without any time restrictions to ensure inter observer reliability. The images were assessed to reach the consensus. A third decisive interpretation was managed by an Endodontist with 11 years of experience in case of any disagreement. To ensure the interrater reliability, the calibration of all evaluators was done by analysis of 20 random cases at different times of a day initially and repeated again after an interval of one week. The Kappa result was interpreted as per Cohen: values ≤ 0 (no agreement), 0.01-0.20 (none to slight agreement), 0.21-0.40 (fair agreement), 0.41-0.60 (moderate agreement), 0.61-0.80 (substantial agreement), and 0.81-1.00 (almost perfect agreement).^12^ The data was tabulated and analysed by utilizing SPSS version 22. The categorical variables were assessed by chi-square test with significance level (p-value) set at < 0.05.

## RESULTS

Out of 314 cases, 232 cases (121 males and 111 females, with a mean age of 37.5 years) accomplished the criteria for inclusion. In these 232 cases, 464 mandibular CI and 464 mandibular LI CBCT images were assessed. All mandibular incisors had one root. The result of the standard consistency check by the evaluators was 0.81, demonstrating reliability of the results in the present study.

**Fig.1:**
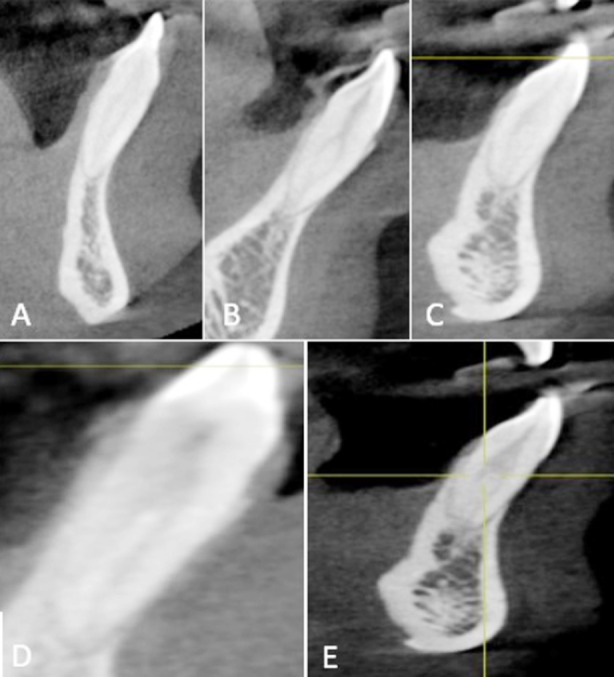
Mandibular incisors CBCT images with A- Type-II, B- Type-III, C- Type-IV, D- Type-V and E- Type-VII.

In 464 mandibular CI, the prevalence of Vertucci Type-I was 70.6% (328 teeth); Type-III was 24.5% (114 teeth); Type-II was 3.4% (16 teeth); Type-IV was 0.9% (4 teeth) and Type-V was 0.5% (2 teeth). Type-VI, VII and VIII were not found. In 464 mandibular LI, the prevalence of Vertucci Type-I was 61.6% (286 teeth); Type-III was 31.8% (148 teeth); Type-II was 4.3% (20 teeth); Type-IV was 0.8% (4 teeth); Type-V was 1.3% (6 teeth) and Type-VII was 0.2% (1 tooth). Type-VI and VIII were not found. (Table-I, Fig.1). The prevalence of more than one canal in mandibular CI was 29.3% and in mandibular LI was 38.57%.

**Table I:** Distribution of various canal anatomies in mandibular incisors. The same columns (uppercase) and rows (lowercase) having dissimilar superscript reveals significant differences.

Tooth	Sample size	Type-I n (%)	Type-II n (%)	Type-III n (%)	Type-IV n (%)	Type-V n (%)	Type-VI n (%)	Type-VII n (%)	Type-VIII n (%)
Mandibular CI	464	328(70.6%)^A,a^	16(3.4%)^A,c^	114(24.5%)^A,b^	4 (0.9%)^A,d^	2(0.5%)^A,d^	0(0%)^A,e^	0(0%)^A,e^	0(0%)^A,e^
Mandibular LI	464	286(61.6%)^B,a^	20(4.3%)^A,c^	148(31.8%)^B,b^	4(0.8%)^A,d^	6(1.3%)^A,c^	0(0%)^A,e^	1(0.2%)^A,e^	0(0%)^A,e^

Total	928	614(66.3%)	36(3.9%)	262(28.3%)	7(0.7%)	8(0.9%)	0(0%)	1(0.1%)	0(0%)

For mandibular CI, significant difference was found between Type-I, II, III and IV canal configurations and Type-I, II, III and V canal configurations (p < 0.05). No significant difference was found between Type-IV and V canal configurations. For mandibular LI, significant difference was found between Type-I, II, III, IV and VII canal configurations (p < 0.05). Type-II and V showed no significant differences. Significant difference was found between mandibular CI and LI in Vertucci Type-I and Type-III canal configurations (p < 0.05).

**Fig.2:**
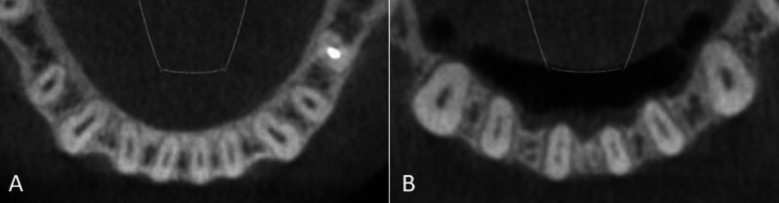
Oval shaped canals at A-3 level in A- Type-I canals in mandibular incisors; and B- Type-III canals in lateral incisors.

Type-VI, VII and VIII canals showed no significant differences. (Table-I) The prevalence of oval shaped canals in Type-I and III canals at apical third level is shown in Table-II. The prevalence of oval canals was significantly higher in Vertucci Type-III canals (80.1%) as compared to Vertucci Type-I canals (32.2%) (p < 0.05) (Fig.2). 46.6% mandibular incisors exhibited oval canals. For both mandibular CI and LI, the prevalence of Type-I canals was significantly higher in males than as compared to females (p < 0.05). However, the prevalence of Type-III canals was significantly higher in females than as compared to males (p < 0.05). There were no significant differences among rest of the canal configurations (Table-III). The prevalence of different canal configurations on the left and the right side of the mouth were not statistically significant (P-value>0.05).

**Table II:** Distribution of oval canals in Type-I and III mandibular incisors in A-3 area. Dissimilar superscript in same column reveals significant difference.

Mandibular Central and Lateral Incisors	n	Oval canals n (%)
Type-I	614	198 (32.2%)^A^
Type-III	262	210(80.1%)^B^
Total n (%)	876	408(46.6%)

**Table III:** Gender-wise distribution of various canal anatomies in mandibular incisors. Dissimilar superscripts (uppercase) in the same column of each type of canal indicate statistically significant difference.

Tooth Type	Gender	n	Type-I n (%)	Type-II n (%)	Type-III n (%)	Type-IV n (%)	Type-V n (%)	Type-VI n (%)	Type-VII n (%)	Type-VIII n (%)
Mandibular CI	Female	222	137(61.7%)^A^	9(4%)^A^	69(31%)^A^	1(0.5%)^A^	2(0.9%)^A^	0(0%)^A^	0(0%)^A^	0(0%)^A^
	Male	242	191(78.9%)^B^	7(2.9%)^A^	45(20.3%)^B^	3(1.2%)^A^	0(0%)^A^	0(0%)^A^	0(0%)^A^	0(0%)^A^
Mandibular LI	Female	222	128(57.6%)^A^	12(5.4%)^A^	86(35.5%)^A^	1(0.4%)^A^	4(1.8%)^A^	0(0%)^A^	1(0.4%)^A^	0(0%)^A^
	Male	242	158(65.2%)^B^	8(3.3%)^A^	62(25.6%)^B^	2(0.8%)^A^	2(0.8%)^A^	0(0%)^A^	0(0%)^A^	0(0%)^A^

The prevalence of more than one canal was significantly higher in 13-30 years age group (58%) than as compared to 31-50 years age group (37.7%) and 51-70 years age group (4.1%) (p < 0.05) (Table-IV).

**Table IV:** Number (n) and percentage (%) of more than one canal in mandibular incisors among different age groups. Different superscript uppercase in the same column indicates statistically significant difference (p < 0.05).

Age (years)	Mandibular incisors with more than one canal n (%)
13-30	183 (58%)^A^
31-50	119 (37.7%)^B^
51-70	13 (4.1%)^C^

## DISCUSSION

Mandibular incisors display wide variations in their root canal morphology. The pulp chamber often contains a dentinal bridge which bifurcates the single canal. In most cases, the two canals join with each other to exit as a single canal, however, sometimes, they may pursue two separate exits resulting in two canals. Infrequently, one canal may branch off into two canals which re-joins again to form a single canal before exiting the apical foramen.^13^

The wide range of variations reported in the literature for a second canal in mandibular incisors has been related to methodologic and racial differences. The presence of additional canals should be perceived to increase the predictability of endodontic success. Thus, the recognition of canal anatomy and its variations is imperative. CBCT is a well-established supplemental technique that provides less radiation, lower scan time and increased accuracy and resolution.^14^ This study used CBCT scans of patients from the database of the Oral and Maxillofacial Radiology department. Thus, the subjects of this study were not exposed to radiation.

In the current study, all mandibular incisors had single root. Vertucci found Type-I canal configuration in 70% mandibular CI and 75% mandibular LI.^15^ Mashyakhy M et al. found the prevalence of Type-I canal in Saudi subpopulation in 73.7% mandibular CI and 69.2% in mandibular LI.^16^ The high prevalence of Type-I canals in mandibular incisors found in this study is in accordance with them. We found the highest prevalence of Type-III canals as an anatomic variation in all mandibular incisors wherein a single canal was present in the cervical third area, divided into two canals in the middle third area and merged to form a single canal again as it exited the apical foramen. Our findings are in corroboration with Al Fouzan et al who used the clearing technique and found that 30% of Type-III canals were present in eighty extracted teeth.^17^

Literature states that the prevalence of two canals in apical third area of mandibular incisors is very low (1-7%) and not commonly seen during routine endodontic diagnosis.^11^,^15^,^18^ Similar to these studies, we found the presence of two canals in the apical third area ranging from 0.2% to 1.3% in mandibular incisors. These findings suggest high prevalence of single canal in the apical third area of mandibular incisors, where proper disinfection can be achieved during routine endodontic therapy as compared to the presence of multiple canals. The presence of a single canal in the apical third area is usually complicated by oval canals. Wu et al defined long oval canals as having a long diameter that is at least twice that of their short diameter.^7^ They found its high prevalence in the apical 5mm. In the present study, we found a high prevalence of oval canals in Type-III (80.1%) and Type-I (32.2%) canals in apical third area. The long oval canals were reported in the mandibular incisors’ middle and coronal thirds, by Shemesh and colleagues. They further noted the presence of 1mm septum in the incisors with two canals, which ultimately merged the two canals into one oval canal.^19^

The real challenge here is the complete disinfection of these long oval canals in the apical third area of mandibular incisors. With the use of rotary, and hand files in oval canals, approximately 66% of the canal wall remains untouched.^20^ With the use of rotary files, a circular preparation is achieved in oval canals. This leaves behind untouched buccal and lingual recesses which in turn harbours bacterial biofilm. Advanced rotary instruments like TRUShape files, and XP-endo Shaper/Finisher files are the preferred instruments for shaping oval canals. Obturation with warm gutta-percha techniques is more efficient in filling such canals.^21^ Hence, it is imperative for the clinician to be aware of these anatomic variations for a successful endodontic outcome.

In females, the prevalence of Type-III canals was significantly higher than in comparison to males. Our findings are in accordance with Geduk et al. who found similar results.^22^ However, Liu et al. found a higher occurrence of the second canal in males than as compared to females.^23^ These differences may be due to alterations in research methods, sample size, ethnicity and genetic constitution. We found no significant difference in the prevalence of different types of canals in mandibular incisors when the left and right sides of the mouth were compared.

Age is of paramount importance for understanding the prevalence of additional canals especially in younger age group. Decrease in the pulp width and narrowing of root canals due to deposition of secondary dentin has been acknowledged with advancing age. This can lead to complete obliteration of root canal in older age groups.^24^ In the present study, we found the prevalence of second canals to be significantly higher in the younger age group of 13-30 years. Our findings are in corroboration with Talabani et al who noted significantly higher prevalence of additional canals in younger age groups as compared to older age groups.^25^

### Limitation of the Study

This study was conducted on a small Saudi sub-population.

### Strength of the Study

In this study, we found a high prevalence of oval shaped canals in mandibular incisors in the Saudi sub-population. This data can help an astute clinician in the selection of appropriate instruments like XP-Endo Shaper (FKG Dentaire, Switzerland) and TRU Shape (Dentsply, Tulsa, OK), that are specifically designed for cleaning/shaping of irregular canals and the use of warm gutta percha techniques for obturating mandibular incisors.

## CONCLUSION

The study found a high prevalence of more than one canal in mandibular central and lateral incisors with Type-III being the prevalent type. Additionally, oval canals were predominantly noted in single canal mandibular incisors, particularly Type-III. Moreover, insignificant difference was noted in the prevalence of different canal configurations on the left and the right sided mandibular incisors. Considering the aforementioned findings, it can be concluded that knowledge of the aberrant canal anatomies may be helpful for the clinician to achieve a favorable endodontic outcome.

### Authors Contribution:

**RAA:** Conceived, designed and did the data collection.

**SS:** Statistical analysis, write up & editing of manuscript.

**MQJ:** Write up, editing, review and final approval of manuscript. MQJ is responsible and accountable for the accuracy and integrity of the work.
